# The effect of continuous ultrasound on chronic non-specific low back pain: a single blind placebo-controlled randomized trial

**DOI:** 10.1186/1471-2474-13-192

**Published:** 2012-10-02

**Authors:** Safoora Ebadi, Noureddin Nakhostin Ansari, Soofia Naghdi, Shohre Jalaei, Mirmostafa Sadat, Hosein Bagheri, Maurits W vanTulder, Nicholas Henschke, Ehsan Fallah

**Affiliations:** 1 Department of physiotherapy, School of Rehabilitation, Tehran University of Medical Sciences, Shahnazari St, Tehran, Iran; 2 Sina Hospital, Medical Faculty, Tehran University of Medical Sciences, Hasanabad St, Tehran, Iran; 3 Department of Health Sciences, VU University, De Boelelaan, Amsterdam, The Netherlands; 4 Musculoskeletal Division NHMRC Postdoctoral Fellow, The George Institute for Global Health, Kent St, Sydney, Australia; 5 Emam Reza hospital, Medical Faculty, Army University of Medical sciences of the I.R.Iran, Etemadzade St., Tehran, Iran

**Keywords:** Low back pain, Ultrasound, Functional disability, Pain, Muscle endurance, Range of motion

## Abstract

**Background:**

Non-specific chronic low back pain (NSCLBP) is one of the most common musculoskeletal disorders around the world including Iran. One of the most widely used modalities in the field of physiotherapy is therapeutic ultrasound (US). Despite its common use, there is still inconclusive evidence to support its effectiveness in patients with NSCLBP. The objective of this study was to evaluate the effect of continuous US compared with placebo US additional to exercise therapy for patients with NSCLBP.

**Methods:**

In this single blind placebo controlled study, 50 patients with NSCLBP were randomized into two treatment groups: 1) continuous US (1 MHz &1.5 W/cm^2)^ plus exercise 2) placebo US plus exercise. Patients received treatments for 4 weeks, 10 treatment sessions, 3 times per week, every other day. Treatment effects were assessed in terms of primary outcome measures: 1) functional disability, measured by Functional Rating Index, and 2) global pain, measured by a visual analog scale. Secondary outcome measures were lumbar flexion and extension range of motion (ROM), endurance time and rate of decline in median frequency of electromyography spectrum during a Biering Sorensen test. All outcome variables were measured before, after treatment, and after one-month follow-up. An intention to treat analysis was performed. Main effects of Time and Group as well as their interaction effect on outcome measures were investigated using repeated measure ANOVA.

**Results:**

Analysis showed that both groups had improved regarding function (FRI) and global pain (VAS) (P < .001). Lumbar ROM as well as holding time during the Sorensen test and median frequency slope of all measured paravertebral muscles did not change significantly in either group (P > .05). Improvement in function and lumbar ROM as well as endurance time were significantly greater in the group receiving continuous US (P < .05).

**Conclusions:**

The study showed that adding continuous US to a semi supervised exercise program significantly improved function, lumbar ROM and endurance time. Further studies including a third group of only exercise and no US can establish the possible effects of placebo US.

**Trial registration:**

NTR2251

## Background

Low back pain (LBP) is a major cause of morbidity in high, middle and low-income countries and affects 80–85% of people over their lifetime [1]. LBP is a common health and socioeconomic problem in Iran. In a cross-sectional study in one of the largest car-manufacturing companies in Iran, the 1-year prevalence of self reported LBP was 21% (20% for males and 27% for females). The prevalence rate of absence due to LBP was 5% per annum [2]. As part of a World Health Organization study, LBP was detected in 15.4% of the population under survey in Tehran (urban area) [3] and in 23.4% of the population in rural areas in Iran [4].

Specific back pain occurs in approximately 2% of all patients with back complaints [5]. For the majority of patients with LBP a specific diagnosis cannot be defined on the basis of anatomical or physiological abnormalities. Non-specific LBP (NSLBP) is assumed to be inflammatory or mechanical in nature [6]. Chronic NSLBP refers to an episode of activity-limiting LBP (with no pain referred into either lower limb) that lasts for 3 months or more [7].

Non-pharmacological methods including a variety of physical agents are the cornerstone of the management of chronic LBP. Therapeutic ultrasound (US) is among the commonly used physical modalities for treating soft tissue injuries [8]. There is a dearth of evidence for the clinical use of therapeutic US in patients with LBP [9].

Therapeutic US is delivered in two modes: 1) Continuous mode in which the delivery of US is non-stop throughout the treatment period; 2) Pulsed mode in which the delivery of US is intermittently interrupted [10].

Therapeutic effects of US are classified as thermal and non-thermal. Ultrasonic energy causes soft tissue molecules to vibrate from exposure to the acoustic wave. This increased molecular motion generates frictional heat and consequently increases tissue temperature. This increased temperature, named thermal effects, is thought to cause changes in nerve conduction velocity, increase in enzymatic activity, changes in contractile activity of skeletal muscles, increase in collagen tissue extensibility, increase in local blood flow, increase in pain threshold, and reducing muscle spasm [11].

Acoustic waves cause normally present minute gas pockets in the tissue to develop into microscopic bubbles or cavities. With therapeutic US, stable acoustic cavitation results, whereby the microbubbles pulsate without imploding. This pulsation leads to microstreaming of fluid around the pulsating bubbles. When occurring around cells, this process, referred to as non-thermal effects, is reported to alter cell membrane activity, vascular wall permeability, and facilitate soft tissue healing [12]. Traditionally, continuous US is used for its thermal effects. Pulsing the US is thought to minimize its thermal effects [10]. In fact, it is not possible to truly isolate the thermal and non-thermal effects as both effects occur with US application [13].

Studies on the efficacy of continuous US in chronic LBP are lacking [8] and there is little evidence of its effectiveness in physiotherapy practice [14,15]. However, lack of evidence is not evidence of lack of effect. Therefore, the main objective of the current study was to compare the effect of continuous US to placebo US combined with exercise therapy on the primary outcomes, functional status and pain of a group of patients with NSCLBP, as well as on the secondary outcomes, endurance of paravertebral and hip muscles, and lumbar range of motion.

## Methods

### Study design

The protocol of this study was approved by the Research Council of Rehabilitation Faculty and the Ethical committee of Tehran University of Medical Sciences (TUMS). The trial was registered with the Netherlands Trial Registry (NTR2251). A more detailed description of the study protocol has been published before [16].

Inclusion criteria in this study were as follows: 1) having NSCLBP, 2) age between 18 and 60. Exclusion criteria were: 1) having nerve root symptoms, 2) having systemic disease and specific conditions such as neoplasm, fractures, spondylolysthesis, spondylolysis, spinal stenosis, ankylosing spondylitis, previous low back surgery, 3) taking medication for specific psychological problems, and 4) being pregnant. Patients were recruited from three university hospitals of TUMS in Tehran, Iran. Patients were provided with oral and written information about the study and were asked to sign a consent form.

#### Sample size

The primary outcome measure of this study was changes in functional status using Functional Rating Index (FRI). Assuming the effect size of .8 for FRI with alpha set at .05 and a power of .8, and accounting for 10% dropouts, the sample size needed was calculated as being 23 patients in each group.

#### Randomization

Randomization was performed using opaque sealed envelopes, which were prepared by a statistician using a computer generated randomization schedule. Half of the envelopes were allocated to each group ensuring equal number of subjects in each group.

### Interventions

The intervention group received continuous US plus semi-supervised exercise; the control group received placebo US plus semi-supervised exercise. Patients were requested not to take pain medications during the intervention period and not to participate in any other exercise or treatment program. All patients in both groups received 10 sessions of treatment, three times a week, every other day.

#### US therapy

Recent reviews of therapeutic US have failed to identify a dose–response relationship [17-19]; though intensities from 0.5 W/cm^2^ to 3 W/cm^2^ have been advocated [18]. Recently published randomized controlled trials, which have reported significant benefits of therapeutic US over placebo US, have used intensities of 1 W/cm^2^ to 1.5 W/cm^2^[20,21].

Mild heating in the chronic phase of injury is known to reduce pain and muscle spasm and to promote healing process. More chronic lesions are treated with continuous US. US frequency of 1 MHz is preferable when treating large and deep soft tissue volumes. Intensities between .8 to 3 W/cm^2^ are suggested for chronic lesions [10,22,23]. Therefore, we chose continuous mode with a frequency of 1 MHz and an intensity of 1.5 W/cm^2^ due to the chronocity of the condition and the deep position of lower back musculature.

US was applied using Enraf Nonius Sonoplus 434, ENRAF,Netherland (coupling gel: Sono Gel, Germany). Slow circular movements were applied using the transducer head over the painful paravertebral low back region. The duration of US was estimated for each patient using Grey’s formula [24]. The average local exposure time was planned to be one minute and the effective radiating area of the transducer head was 5 cm^2^. For a patient with an area of low back pain of 40 cm^2^, for example, the required total treatment time was: 1 min × (40 cm^2^/5 cm2) = 8 minutes.

Patients in the intervention group received continuous US. Placebo US was delivered according to Hashish et al. [25]. The therapist moved the applicator at the same rate and pressure as for the continuous US group. The machine and the light-emitting diode which signaled that its power was connected were in view of the subject, but the dials which indicated the US were out of sight. Commonly, the patient is not aware of what she/he should expect at the beginning of treatment with US and since even with real US subjects are unaware of any sensation at most therapeutic intensities [22], patients were told in both groups that they may feel some heat and should this cause discomfort, to notify the therapist in order to safeguard patients in the continuous US group from overheating.

#### Exercise therapy

There is strong evidence that exercise is as effective as other conservative treatments in chronic LBP, and functional and pain outcomes significantly improves in groups receiving exercises relative to other interventions [26]. Studies indicate that stretching and strengthening exercises can improve pain and function. Home exercises combined with therapist supervision have been identified as the most effective strategy for patients with CLBP [27].

It is recognized that the abdominal muscles, back extensors, and gluteals are weak in patients with CLBP, which can cause significant spinal loading. Patients with LBP also exhibit tightness of hamstring and hip extensors, which may impair spinal mechanics. Therefore, strengthening and flexibility exercises are important for a healthy lower back [28].

A semi-supervised exercise program was developed. The program included posterior pelvic tilts, sit-ups, bridging, quadruped exercises, and posterior hip and knee muscles stretching [29,30]. Patients were instructed to perform 2 to 3 stretches (of all muscles) per day and hold the stretch for 20 seconds unless it hurts. Strengthening exercises started with 5 repetitions and progressed according to each patient’s improvement, to 3 sets of 10 repetitions. Patients received a pamphlet describing exercises with figures. To emphasize correct performance of the exercises at home, all exercises were checked by the therapist on each treatment session.

Patients were asked to perform the exercises daily; the stretching exercises before the strengthening exercises. They were advised to stay active during the day, and walk for at least 15 minutes before exercising, which could also act as a warm-up. After completion of all treatment sessions, patients were asked to maintain the daily home exercises for one further month. During the period from the completion of the treatment to the follow-up measuring session (1 month), patients visited the clinic once a week to control their exercises for correct performance.

### Outcome measures

Primary and secondary outcome measures were documented at baseline, after the final treatment session (after 4 weeks), and at one-month follow-up.

Pain and function are the two most fundamental clinical outcomes for low back pain [31], while accurate assessment of lumbar range of motion has been recommended as a core domain in the evaluation of patients with lumbar dysfunction and monitoring treatment progress [32,33]. Since the endurance of trunk muscles has been shown to be related to the incidence of low back pain, surface electromyography, specifically power spectral analysis of EMG signals has become an increasingly common method for the assessment of lumbar muscle activity and localized muscle fatigue and has been suggested as an objective, safe, easy and non invasive measure for the evaluation of patients with low back pain [34].

Readers are referred to the design article of this study for further details on assessment methods related to outcome measurements [16].

The Primary outcomes were functional disability measured by the Persian version of the Functional Rating Index (FRI) [35-37] and pain intensity measured during last week on a 100 mm visual analogue scale (VAS) [38].

Secondary outcome measures were paravertebral muscle fatigue during a Biering-Sorensen test using surface electromyography [39], and lumbar flexion and extension range of motion using the Modified-Modified Schober Test (MMST) [40].

Briefly, electromyographic data acquisition was performed using an 8-channel surface EMG recorder (DATA Log Biometrics Ltd) and analyzed by the built in software, DATA LOG PC software version 7.5 (Biometrics Ltd, UK). The software applied Fast Fourier transformation to calculate median frequency and gave the rate of decline in median frequency (MF slope) by trend lines which were calculated using Linear Regression Analysis based upon the least squares method to produce a slope **m** and an intercept of the Y-axis. Preamplified bipolar Ag-AgCl electrodes (Type NO.SX230, Biometrics Ltd, UK, 10 mm in diameter) with fix center to center inter electrode distance of 20 mm were used. The signal was gathered at a sample rate of 1000 Hertz and a gain of 1000 Decibel.

### Data analysis

All data were analyzed using SPSS V19, SPSS Inc.,Chicago, IL, USA. Kolmogorov-smirnov test revealed normal distribution of data. Repeated measure ANOVA was used to determine the main and interaction effects of Time and Group on the outcome measures. Bonferroni test was used for pos -hoc analysis when necessary.

An intention-to-treat analysis of the data was performed to retain data for all patients. In the case of dropouts, the last recorded values for the outcome measures were used in the analysis (Last Observation Carried Forward (LOCF)). *p*-values ≤ .05 were considered as statistically significant.

## Results

**Figure 1:**
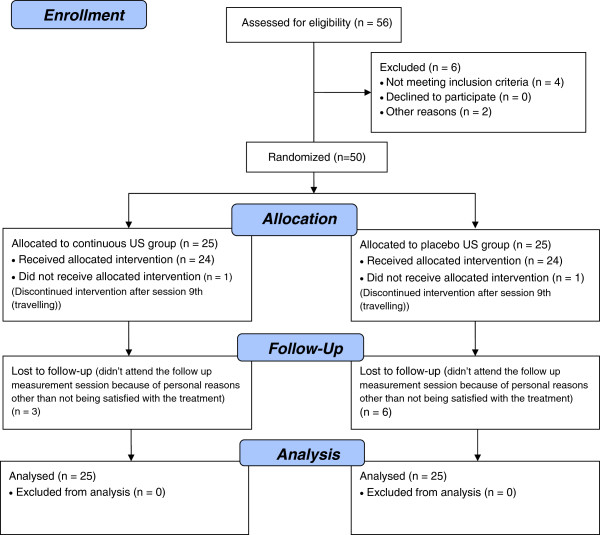
Flow diagram of participation and withdrawals for patients in continuous ultrasound and placebo ultrasound groups.

Figure 1 shows a flow chart of participants. A total of 50 patients were randomized, 25 to each group. One patient in each group dropped out after the 9^th^ session, because of personal reasons. Nine more patients (3 patients in the experimental group and 6 patients in the placebo group) did not complete the follow-up measurement, because of travelling, complete improvement or other personal reasons.

Mean age of all participants was 34.7 (SD 12.6) years with a mean pain history of 7.0 (SD 4.6) years. There was no statistically significant difference in baseline characteristics as well as baseline outcome measures between groups except for endurance time (Table 1). Possible effect of endurance time at baseline was measured by evaluating the effect of adding this variable to the model using analysis of covariance (ANCOVA). It resulted in no change in the statistical significance of the Time or Group effects.

**Table 1:** Characteristics of patients with non specific chronic low back pain before treatment in continuous ultrasound and placebo ultrasound groups

**Parameter**	Continuous US*		**Placebo US**		P value***
	**(n=25)**		**(n=25)**		
	**Mean**	**SD**	**Mean**	**SD**	
Age(years)	31.4	12.3	37.4	11.9	.09
Onset since first episode(years)	5.8	4.1	8.1	4.7	.08
BMI**	24.4	4.1	25.3	3.5	.39
Sex	25%female _ 75%male		50%female _ 50%male		

* Ultrasound.

** Body Mass Index.

***p values are for baseline differences between the two groups. Significance level ≤ .05.

Mean values for baseline, after 10 session treatment and 1-month follow up measurements as well as P values for baseline differences are shown in Table 2. As can be seen, FRI has shown improvement (decreased) in both groups. Also, VAS scores have dropped in both groups. Details of secondary outcome measures can be found in Table 2.

**Table 2:** Mean and SD of primary and secondary outcome measures for continuous ultrasound and placebo ultrasound groups at baseline, after 10 treatment sessions, and after 1 month follow up

**Parameter**	Continuous US*			**Placebo US**			P value***
	**Before treatment**	**After 10 sessions**	**After 1 month**	**Before treatment**	**After 10 sessions**	**After 1 month**	
	**N=24**	**N=24**	**N=21**	**N=24**	**N=24**	**N=18**	
FRI**	40.8 (14.6)	23.4 (6.9)	22.8 (7.8)	43.9 (16.9)	31.1 (13.4)	30.5 (11.9)	.49
VAS**	46.6 (17.7)	26.6 (13.8)	27.7 (14.4)	49 (16)	30.7 (13.1)	25.5 (9.9)	.62
Flexion ROM (millimeters)	48.8 (19.4)	52.4 (18.6)	52.4 (19.60)	57.4 (18.9)	59.8 (17.9)	57.5 (18.3)	.13
Extension ROM (millimeters)	19.4 (8.2)	20.12 (8.5)	21.7 (8.5)	23.6 (9.6)	24.1 (9.3)	24.7 (9.6)	.11
Endurance time(in seconds)	111.5 (33.5)	128.9 (30.2)	128.3 (26.2)	134.2 (27.1)	140.3 (43.5)	139.3 (45.8)	.01
Median frequency slope of right muscles							
Illiocostalis lumborum	-.24 (.17)	-.21 (.09)	-.19 (.07)	-.21 (.13)	-.20 (.06)	-.19 (.06)	.56
Multifidus	-.26 (.15)	-.26 (.16)	-.24 (.13)	-.30 (.17)	-.25 (.05)	-.24 (.05)	.82
Gluteus maximus	-.11 (.13)	-.09 (.10)	-.09 (.10)	-.13 (.13)	-.09 (.09)	-.09 (.1)	.65
Biceps femoris	-.12 (.09)	-.12 (.08)	-.09 (.06)	-.11 (.07)	-.12 (.07)	-.09 (.05)	.83
Median frequency slope of left muscles							
Illiocostalis lumborum	-.18 (.11)	-.18 (.11)	-.16 (.09)	-.21 (.12)	-.19 (.07)	-.18 (.08)	.29
Multifidus	-.24 (.14)	-.25 (.15)	-.25 (.15)	-.29 (.21)	-.24 (.10)	-.25 (.11)	.31
Gluteus Maximus	-.06 (.04)	-.06 (.06)	-.08 (.05)	-.09 (.07)	-.08 (.05)	-.09 (.08)	.11

*US: Ultrasound.

**FRI: Functional Rating Index, VAS: Visual Analog Scale.

***p values are for baseline differences between the two groups at significance level ≤ .05.

Table 3 details the results of the mixed model ANOVA [Group (US and placebo US) × Time (Pre, Post, and follow-up)] showing the effect of continuous US versus sham US on outcome measures.

**Table 3:** Main effects of Time and Group and their interaction effect on primary and secondary outcome measures (CI=95%,) *

**Outcome measure**	**Effects**	**df**	**F**	**P value**
FRI**	**Time**	2	75.92	<.001*
	**Group**	1	3.90	.004*
	Time*Group	2	1.03	.31
VAS**	**Time**	2	80.11	<0.001*
	**Group**	1	.00	.48
	Time*Group	2	.514	.98
**Lumbar flexion**	**Time**	2	3.47	.09
	**Group**	1	3.16	.02*
	Time*Group	2	1.48	.23
**Lumbar extension**	**Time**	2	1.53	.11
	**Group**	1	4.12	.03*
	Time*Group	2	1.61	.21
**Endurance time**	**Time**	2	0.63	.43
	**Group**	1	3.05	.05*
	Time*Group	2	1.99	.17
**Right Illiocostalis Lumborum**	**Time**	2	.39	.53
	**Group**	1	.01	.93
	Time*Group	2	.52	.41
**Right Multifidus**	**Time**	2	.06	.16
	**Group**	1	.16	.69
	Time*Group	2	.52	.48
**Right Gluteus Maximus**	**Time**	2	3.41	.73
	**Group**	1	.02	.89
	Time*Group	2	.52	.47
**Right Biceps Femoris**	**Time**	2	5.38	.86
	**Group**	1	.02	.79
	Time*Group	2	.05	.82
**Left Iliocostalis** **Lumborum**	**Time**	2	3.65	.06
	**Group**	1	.87	.35
	Time*Group	2	.38	.54
**Left Multifidus**	**Time**	2	1.49	.23
	**Group**	1	.14	.71
	Time*Group	2	1.22	.27
**Left Gluteus Maximus**	**Time**	2	.98	.33
	**Group**	1	.95	.33
	Time*Group	2	.86	.36

*The effect of the US versus placebo US on outcome measures was analyzed using a Group (US and placebo US) × Time (Pre, Post, and follow-up) mixed model ANOVA at significance level ≤ .05.

**FRI: Functional Rating Index, VAS: Visual Analog Scale.

### Primary outcome measures

There was a significant effect of Time (p < .001) on FRI. Bonferroni post-hoc test revealed that FRI scores had improved significantly after 10 treatment sessions (p < .001) and over time after one month follow-up in both groups (p < .001). The improvement of FRI scores was maintained one month after the end of the 10^th^ treatment session (p = .24). Main effect of Group on FRI was significant (p = .004) while the Time × Group interaction was not significant (p = .31).

There was a significant effect of Time on VAS (p < .001). The mixed model ANOVA on VAS did not reveal a statistically significant Group effect (p = .48). Post-hoc analysis showed that VAS scores improved significantly from baseline to after the 10^th^ session (p < .001) and continued to improve until the one-month follow-up measurement (p = .004). The Time × Group interaction was not significant (p = .48).

### Secondary outcome measures

Main effect of Time was not significant on both flexion (p = .09) and extension lumbar ROM (p = .11). However, a significant Group effect was identified for flexion (p = .02) and extension (p = .01). The interaction effect of Time × Group on lumbar range of motion was not significant (flexion: p = .23, extension: p = .21).

The values for median frequency slope did not show a statistically significant Time effect (p > .05); Group effect (p > .05) or Group × Time interaction (p > .05).

Although main effect of Time on holding time during Sorensen test was not significant (p = .09), the effect of Group showed statistical significance (p = .01). There was no interaction effect of Time × Group on this parameter.

## Discussion

In everyday clinical practice the application of US is often combined with other physiotherapeutical interventions, usually with exercise therapy [41]. The aim of this study was to investigate whether continuous US can add to the effects of exercise therapy in patients suffering from NSCLBP compared to placebo US.

The results showed that both FRI and VAS have improved after 10 sessions of treatment and over time after 1 month in both groups. FRI improvement was significantly greater in the group receiving continuous US. This finding is consistent with Ansari et al. [20] who demonstrated a better functional outcome in a continuous US group in comparison with a placebo US group. In their study patients did not receive any treatment in addition to continuous and placebo US. Other randomized trials in which the effect of US is directly compared with placebo US in NSCLBP are lacking. US is usually studied in comparison with other modalities [42,43] or is presented in a package of physiotherapy [44] and is also investigated in other subgroups of patients with LBP other than non-specific LBP, such as lumbar disk herniation [45-47].

Durmus et al. [42] in comparing 3 groups of NSCLBP patients who received US + exercise, Electrical Stimulation (ES) + exercise and exercise only, showed significantly greater improvement in pain and function of the ES and US groups in comparison with the control group. The study found no difference in function between groups receiving either ES, or US but the US group had significantly better scores regarding pain improvement.

Mohseni et al. [43] compared manipulation and exercise treatment with US and exercise treatment in a randomized clinical trial. One hundred and twenty patients with chronic LBP were given a program of exercises. In addition, one group received spinal manipulation therapy and the other group received therapeutic US. Pain intensity, functional disability, lumbar movements measured by Modified Modified Schober Test and muscle endurance were measured shortly before treatment, at the end of the treatment program and 6 months after randomization using surface electromyography. Although improvements were recorded in both groups, patients receiving manipulation/exercise showed a greater improvement compared with those receiving US/exercise at both the end of the treatment period and at 6-month follow-up. The authors did not report on the details of the exercise program, and US delivery was inconsistent (continuous 1MHz, 1.5-2.5W/cm^2^ for 5 to 10 minutes, average 6 sessions, one or two times a week) which could both be possible sources of difference with our study.

Since the current study lacks a third group with no US, it is impossible to explore the effects of exercise and US separately except in parts where the continuous US group has shown significant differences in comparison to the placebo group. As both groups in our study improved significantly regarding pain, we can conclude that the treatment common to both groups (exercise and mechanical application of US head) have attributed to the outcome. There is strong evidence that exercise is an effective treatment in chronic low-back pain [48]. Exercise programs for CLBP may be designed to reverse deconditioning or the fear of movement associated with pain. Such exercises typically include aerobic exercises like walking as well as strengthening and stretching regimens [27]. The specific exercises administered to patients in this study may have been of benefit in improving pain. Since many items of the FRI questionnaire are indirectly related to the pain experienced by patients during that specific task, the decreased pain achieved with treatment could have caused the patients in both groups to perform better during those tasks as well.

However, the individual role of the placebo effects of US in the placebo group as well as the individual effect of mechanical movement of US head and exercising in both groups cannot be specified although each one may have played a part in the outcome. A placebo effect of US can be the result of moving the applicator head thus benefitting from the effects of massaging [20,25]. Continuous movement of the applicator may increase the temperature of the area under treatment and may stimulate the skin receptors causing the pain gate control mechanism to become active [20]. It has been shown that moving the applicator of US on the affected area can change the level of serum cortisol, which in turn can affect inflammation and swelling [25]. Patients in both groups could have benefitted from the Placebo effects of the treatment [49].

A significant difference in the improvement of FRI scores in favor of the continuous US group can be related to the thermal and mechanical effects of continuous US.

Morrisette et al. [50] showed that continuous 1 MHz US given at either 1.5 W/cm^2^ or 2.0 W/cm^2^ intensity has the capability of heating lumbar periarticular tissue while the intervening muscle may heat as well. Morrisette stated that the temperature elevation was at a level thought to be sufficient to produce the theoretical therapeutic effects proposed with an elevation in temperature.

Regarding secondary outcome measures, although lumbar flexion and extension ROM increased in both groups after treatment, the increase did not reach statistical significance within groups. Nevertheless, the amount of improvement in ROM was significantly greater in the continuous US group. Durmus et al. [42] reported significant improvement in Modified Schober scores in the group receiving US + exercise. However, this improvement was not significantly different from the two other treatment groups receiving ES + exercise and exercise only. In the study carried out by Mohseni et al. [43], lumbar flexion and extension ROM as measured by MMST (Modified Modified Schober Test) improved significantly in US + exercise group but this improvement was significantly lower in comparison with the manipulation + exercise group. Though none of the studies above, had reported the exact exercises prescribed, their difference with our study can be possibly explained by the differences in exercise type and intensity and patient population as well as the difference in the dosage of US.

Clinical assessment of movement impairment in low back pain is predominantly done by measuring changes in lumbar ROM in order to investigate patient’s response to treatment [51]. The reduction in pain alongside stretching and strengthening exercises prescribed could have contributed to the increase of ROM in both groups. The significant additional increase of ROM in the continuous US group may be due to the thermal and mechanical effects of continuous US. It has been shown that temporary increases in range of movement can be produced by US treatment [52]. There is considerable evidence that the extensibility of collagen based tissues will change with ultrasound thermal applications as long as sufficient temperature change is achieved [53]. Since the therapeutic window for stretching following US application is limited to some 3 minutes immediately after treatment [54], our participants that performed exercises after the treatment sessions, at home, barely could have benefited from such thermal effect. Given that patients suffering from chronic low back pain usually have spasm [7], using continuous US could have been effective in decreasing spasm [10] and consequently resulting in greater ROM increase in comparison with placebo US.

Considering surface EMG parameters, no significant effect of Time or Group was found on median frequency slope of all measured muscles.

The assessment of fatigue based on SEMG techniques during a fatiguing contraction can be demonstrated by a trend of the power spectrum to lower frequencies usually measured by the decrease in median frequency. It has been proposed that better endurance would exhibit a less precipitous decay rate of the median frequency [55], though conflicting opinions exist [56]. It has been indicated that trunk muscle endurance can be increased by using specific exercises [57].

Sung [58] investigated changes in multifidi muscle endurance and functional status after a 4-week supervised spinal stabilization exercise program in 16 patients presenting with chronic low back dysfunction (LBD). Results showed that Oswestry scores improved significantly from pre to post treatment. Significant pre- to post treatment increase in multifidi muscle fatigue for men coupled with a nonsignificant improvement in multifidi muscle endurance for women was also seen. Sung [58] concluded that a 4-week spinal stabilization exercise program significantly improved functional status in patients presenting with LBD but the program was insufficient to effect muscle fatigue. In another study, Mohseni et al. [43] did not find any significant change in median frequency slope or endurance time in the group of patients with low back pain who received continuous US plus exercise for an average of 6 sessions. We also witnessed a nonsignificant change in MF slope of measured paravertebral muscles, which may imply that the usefulness and sensitivity of this parameter was limited in our study.

Regarding endurance time, the group receiving continuous US showed a significantly greater increase than the placebo group. Traditionally, endurance is thought of as the time for sustaining a nonstationary activity, which ceases with fatigue [59]. One of the main reasons for muscle fatigue is the accumulation of metabolite wastes in the region and the inability of the system to provide adequate blood circulation to supply oxygen to the tissue and deplete it from wastes [60]. Additionally, ischemia due to inflammation and spasm is a common finding in chronic low back pain [7,28,61]. It is possible that continuous US has improved low back muscle fatigue by increasing blood circulation in the region and helping improve blood supply [17,23,61] which in turn have caused more sufficient and longer muscle contraction during the test.

### Limitations

The main limitation of this study could be that the treating physiotherapist who collected the data was not blinded to the group allocation. The number of dropouts in our study was higher than what we had predicted at 1 month (22%). The self-reported compliance rate seemed high, but it was not checked. The study lacks a third group without US which makes it impossible to comment on individual interventions separately.

## Conclusions

This single blind, placebo -controlled, randomized clinical trial showed that adding 1 MHz, 1.5 W/cm^2^ US to a semi-supervised regimen of exercise had significantly beneficial effects on function, lumbar flexion and extension ROM, and endurance time in patients with NSCLBP.

Further studies including a third group of no US are needed to explore the differential effects of each intervention on patients with NSCLBP. In addition, it would be helpful to measure other surface electromyography parameters other than median frequency slope, such as mean frequency, initial median frequency and normalized median frequency slope to explore the possible effects of the method used in this study on these parameters.

Studies, in which the methodological shortcomings of this study and similar studies are addressed, are needed to verify a dose response relation in patients with chronic low back pain.

## Competing interests

The authors declare that they have no competing interests.

## Authors' contributions

SE and NNA came up with the original concept for the study. SN, NH, and MvT helped to design the study and contributed to the development of the manuscript. EF and MS coordinated and referred the patients, HB participated in the executive steps of the study and SHJ performed the statistical analysis. SE wrote the first draft of the manuscript with help from the other authors. All authors read and approved the final manuscript.

## Pre-publication history

The pre-publication history for this paper can be accessed here:

http://www.biomedcentral.com/1471-2474/13/192/prepub

## References

[B1] Hoy D March L Brooks P Woolf A Blyth F Vos T Buchbinder R Measuring the global burden of low back pain Best Pract Res Cl Rh 2010 24 155 165 10.1016/j.berh.2009.11.002 20227638

[B2] Ghaffari M Alipour A Jensen I Farshad AA Vingard E Low back pain among Iranian industrial workers Occup Med (Lond) 2006 56 455 460 10.1093/occmed/kql062 16837536

[B3] Davatchi F Jamshidi AR Banihashemi AT Gholami J Forouzanfar MH Akhlaghi M Barghamdi M Noorolahzadeh E Khabazi AR Salesi M WHO-ILAR COPCORD Study (Stage 1, Urban Study) in Iran J Rheumatol 2008 35 1384 18464299

[B4] Davatchi F Tehrani Banihashemi A Gholami J Faezi ST Forouzanfar MH Salesi M Karimifar M Essalatmanesh K Barghamdi M Noorolahzadeh E The prevalence of musculoskeletal complaints in a rural area inIran: a WHO-ILAR COPCORD study (stage 1, rural study) in Iran Clin Rheumatol 2009 28 1267 1274 10.1007/s10067-009-1234-8 19629618

[B5] Verbunt JA Seelen HA Vlaeyen JW van de Heijden GJ Heuts PH Pons K Knottnerus JA Disuse and deconditioning in chronic low back pain: conceptsand hypotheses on contributing mechanisms Eur J Pain 2003 7 9 21 10.1016/S1090-3801(02)00071-X 12527313

[B6] Walker BF Williamson OD Mechanical or inflammatory low back pain. What are the potential signs and symptoms? Manual Ther 2009 14 314 320 10.1016/j.math.2008.04.003 18555728

[B7] Waddell G The Back Pain Revolution 2004 Churchill-Livingstone, London

[B8] Grazio S Markulinèiæ B Nemèiæ T Grubisic F Matijeviæ V Skala H Kasun B Koprivnjak V Trgovec: Effect of intereferential current and therapeutic ultrasound on lumbar spine range of motion in patients with chronic low back pain Proceedings of the 7th Mediterranean Congress of Physical and Rehabilitation Medicine: 18–21 September 2008 Portoro (Slovenia)

[B9] Koes BW van Tulder M Lin CW Macedo LG McAuley J Maher C An updated overview of clinical guidelines for the management of non-specific low back pain in primary care Eur Spine J 2010 19 12 2075 2094 10.1007/s00586-010-1502-y 20602122 PMC2997201

[B10] Cameron M Physical Agents in Rehabilitation: From Research to Practice 2003 Saunders, St. Lous, Missouri

[B11] Chan AK Myrer JW Measom GJ Draper DO Temperature Changes in Human PatellarTendon in Response to TherapeuticUltrasound J Athl Training 1998 33 2 130 135 PMC1320399 16558499

[B12] Allen RJ Physical agents used in the management of chronic pain by physical therapists Phys Med Rehabil Clin N Am 2006 17 315 345 10.1016/j.pmr.2005.12.007 16616270

[B13] Gallo JA Draper DO Brody LT Fellingham GW A Comparison of Human MuscleTemperature Increases During 3-MHzContinuous and Pulsed Ultrasound WithEquivalent Temporal Average Intensities J Orthop Sport Phys 2004 34 395 401 10.2519/jospt.2004.34.7.395 15296367

[B14] Airaksinen O Brox JI Cedraschi C Hildebrandt J Klaber-Moffett J Kovacs F COST B13 Working Group on Guidelines for Chronic Low Back Pain. Chapter 4. European guidelines for the management of chronic nonspecific low back pain Eur Spine J 2006 15 Suppl 2 192 300 10.1007/s00586-006-1072-1 PMC3454542 16550448

[B15] NICE National Institute for Health and Clinical Excellence. Low back pain: Early management of persistent non-specific low back pain. Clinical guideline 88 2009 www.nice.org.uk/cg88

[B16] Ebadi S Ansari NN Henschke N Naghdi S van T The effect of continuous ultrasound on chronic low back pain: protocol of a randomized controlled trial BMC Musculoskelet Disord 2011 12 59 10.1186/1471-2474-12-59 21406117 PMC3069953

[B17] Robertson VJ Dosage and treatment response in randomized clinical trials of therapeutic ultrasound Phys Ther Sport 2002 3 124 133

[B18] van der Windt DA van der Heijden GJ van den Berg SG ter Riet G de Winter AF Bouter LM Ultrasound therapy for musculoskeletal disorders: a systematic review Pain 1999 81 257 271 10.1016/S0304-3959(99)00016-0 10431713

[B19] Laakso EL Robertson VJ Chipchase LS The place of electrophysical agents in Australian and New Zealand entry-level curricula: is there evidence for their inclusion? Aust J Physiother 2002 48 251 254 12443519 10.1016/s0004-9514(14)60164-1

[B20] Ansari NN Ebadi S Talebian S Naghdi S Mazaheri H Olyaei G Jalaie S A randomized, single blind placebo controlled clinical trial on the effect of continuous ultrasound on low back pain Electromyogr Clin Neurophysiol 2006 46 329 336 17147074

[B21] Durmuz D Akyol Y Cengiz K Terzi T Cantürk F Effects of Therapeutic Ultrasound on Pain, Disability, Walking Performance, Quality of Life, and Depression in Patients with Chronic Low Back Pain: A Randomized, Placebo Controlled Trial Turk J Rheumatol 2010 25 82 87 10.5152/tjr.2010.07

[B22] Robertson V Ward A Low J Reed A Electrotherapy Explained Principles and Practice 2007 Butterworth Heinemann, London

[B23] Blanger AY Therapeutic Electrophysical Agents: Evidence Behind Practice. Therapeutic Electrophysical Agents: Evidence Behind Practice 2010 Lippincott Williams & Wilkins, Philadelphia

[B24] Grey K Distribution of Treatment Time in Physiotherapeutic Application of Ultrasound Physiotherapy 2003 89 696 707 10.1016/S0031-9406(05)60492-0

[B25] Hashish I Hai HK Harvey W Feinmann C Harris M Reduction of postoperative pain and swelling by ultrasound treatment: a placebo effect Pain 1988 33 3 303 311 10.1016/0304-3959(88)90289-8 3419838

[B26] Hayden J MW V-T Malmivaara A Koes BW Exercise therapy for treatment of non-specific low back pain Cochrane Database of Systematic Reviews 2005 Issue 3. Art. No.: CD000335 10.1002/14651858.CD000335 PMC10068907 16034851

[B27] Hayden JA van Tulder MW Malmivaara AV Koes BW Meta-analysis: exercise therapy for nonspecific low back pain Ann Inter Med 2005 142 765 775 10.7326/0003-4819-142-9-200505030-00013 15867409

[B28] Vleeming A Mooney V Stoeckart R Movement, Stability & Lumbopelvic pain, integration of research and therapy 2007 Churchill Livingstone, London

[B29] Hall CM Brody LT Therapeutic exercise. Moving toward function 1999 Lippincott Williams & Wilkins, Philadelphia

[B30] Hurtling D Kessler RM Management of common musculoskeletal disorders: physical therapy principles and methods 2006 Lippincott Williams & Wilkins, Philadelphia

[B31] Ostelo RW Deyo RA Stratford P Waddell G Croft P Von Korff M Bouter LM de Vet HC Interpreting change scores for pain and functional status in low back pain: towards international consensus regarding minimal important change Spine 2008 33 1 90 94 10.1097/BRS.0b013e31815e3a10 18165753

[B32] van der Heijde D Bellamy N Calin A Dougados M Khan MA van der Linden S Preliminary core sets for endpoints in ankylosing spondylitis. Assessments in Ankylosing Spondylitis Working Group J Rheumatol 1997 24 11 2225 2229 9375888

[B33] Fritz JM Piva SR Physical impairment index: reliability, validity and responsiveness in patients with acute low back pain Spine 2003 28 11 1189 1194 12782991 10.1097/01.BRS.0000067270.50897.DB

[B34] Mohseni-Bandpei M Watson MJ Richardson B Application of Surface Electromyography in the Assessment of Low Back Pain: A Literature Review Phys Ther Rev 2000 5 2 93 105 10.1179/108331900786208860

[B35] Feise RJ Menke JM Functional rating index: a new valid and reliable instrument to measure the magnitude of clinical change in spinal conditions Spine 2001 26 78 86 10.1097/00007632-200101010-00015 11148650

[B36] Feise RJ Menke JM Functional Rating Index: literature review Med Sci Monit 2010 16 25 36 20110929

[B37] Ansari NN Feise RJ Naghdi S Ebadi S Yoosefinejad AK The Functional Rating Index: Reliability and Validity of the Persian Language Version in Patients with Low Back Pain Spine 2011 36 24 1573 1577 10.1097/BRS.0b013e3182103282 21270679

[B38] Ogon M Krismer M Söllner W Kantner-Rumplmair W Lampe A Chronic low back pain measurement with visual analogue scales in different settings Pain 1996 64 3 425 428 10.1016/0304-3959(95)00208-1 8783305

[B39] Biering-Sorensen F Physical measurements as indicators for low back trouble over a one year period Spine 1984 9 106 119 10.1097/00007632-198403000-00002 6233709

[B40] Williams R Binkley J Bloch R Goldsmith CH Minuk T Reliability of the modified-modified Schober and double inclinometer methods for measuring lumbar flexion and extension Phys Ther 1993 73 33 44 8417457

[B41] van der Windt DA van der Heijden GJ van den Berg SG ter Riet G de Winter AF Bouter LM Ultrasound therapy for musculoskeletal disorders: a systematic review Pain 1999 81 257 271 10.1016/S0304-3959(99)00016-0 10431713

[B42] Durmus D Durmaz Y Canturk F Effects of therapeutic ultrasound and electrical stimulation program on pain, trunk muscle strength, disability, walking performance, quality of life, and depression in patients with low back pain: a randomized-controlled trial Rheumatol Int 2009 30 901 910 19644691 10.1007/s00296-009-1072-7

[B43] Mohseni-Bandpei MA Critchley J Staunton T Richardson B A prospective randomised controlled trial of spinal manipulation and ultrasound in the treatment of chronic low back pain Physiotherapy 2006 92 34 42 10.1016/j.physio.2005.05.005

[B44] Dogan SK Tur BS Kurtaise Y Atay MB Comparison of three different approaches in the treatment of chronic low back pain Clin Rheumatol 2008 27 873 881 10.1007/s10067-007-0815-7 18188660

[B45] Gnatz M Increased radicular pain due to therapeutic ultrasound applied to the back Arch Phys Med Rehab 1989 70 493 494 10.1016/0003-9993(89)90014-2 2525018

[B46] Nwuga VCB Ultrasound in the treatment of back pain resulting from prolapsed intervertebral disc Arch Phys Med Rehabil 1983 64 88 89 6218793

[B47] Unlu Z Tascl S Tarhan S Pabuscu Y Islak S Comparison of 3 physical therapy modalities for acute pain in lumbar disc herniation measured by clinical evaluation and magnetic resonance imaging J Manip Physiol Ther 2008 31 191 198 10.1016/j.jmpt.2008.02.001 18394495

[B48] van Middelkoop M Rubinstein SM Kuijpers T Verhagen AP Ostelo R Koes BW van Tulder MW A systematic review on the effectiveness of physical and rehabilitation interventions for chronic non-specific low back pain Eur Spine J 2011 20 19 39 10.1007/s00586-010-1518-3 20640863 PMC3036018

[B49] Richardson PH Placebo effects in pain management Pain Rev 1994 1 15 32

[B50] Morrisette DC Brown D Saladin ME Temperature Change in Lumbar Periarticular Tissue With Continuous Ultrasound J Orthop Sport Phys 2004 34 754 760 10.2519/jospt.2004.34.12.754 15643730

[B51] Intolo P Milosavljevic S Baxter DG Carman AB Pal P Munn J The effect of age on lumbar range of motion: A systematic review Man Ther 2009 14 6 596 604 10.1016/j.math.2009.08.006 19729332

[B52] Knight CA Rutledge CR Cox ME Acosta M Hall SJ Effect of Superficial Heat, Deep Heat, and ActiveFlexors Exercise Warm-up on the Extensibility of the Plantar Phys Ther 2001 81 1206 1214 11380276

[B53] Meakins A Watson T Longwave ultrasound and conductive heating increase functional ankle mobility in asymptomatic subjects Phys Ther Sport 2006 7 74 80 10.1016/j.ptsp.2005.11.006

[B54] Draper DO Ricard MD Rate of temperature decay in human muscle following 3 MHz ultrasound: the stretching window revealed J Athl Training 1995 30 4 304 307 PMC1317998 16558352

[B55] Mannion AF Junge A Taimela S Müntener M Lorenzo K Dvorak J Active therapy for chronic low back pain: part 3. Factors influencing self-rated disability and its change following therapy Spine 2001 26 920 929 10.1097/00007632-200104150-00015 11317114

[B56] Elfving B Dedering A Nemeth G Lumbar muscle ratigue and recovery in Patients with long-term low-back trouble- electromyoghraphy and health related factors Clin Biomech 2003 18 619 630 10.1016/S0268-0033(03)00095-0 12880709

[B57] Jorgensen K Nicolaisen T Trunk extensor endurance: determination and relation to low-back trouble Ergonomics 1987 30 259 267 10.1080/00140138708969704 2953591

[B58] Sung PS Multifidi muscles median frequency before and after spinal stabilization exercises Arch Phys Med Rehabil 2003 84 9 1313 1318 10.1016/S0003-9993(03)00139-4 13680567

[B59] Moffroid MT Haugh LD Haig AJ Henry SM Pope MH Endurance training of trunk extensor muscles Phys Ther 1993 73 10 17 8417455

[B60] Williams CA Ratel S Human Muscle Fatigue 2009 Routledge

[B61] Nadler SF Weingand K Kruse RJ The Physiologic Basis and Clinical Applications of Cryotherapy and Thermotherapy for the Pain Practitioner Pain Physician 2004 7 395 399 16858479

